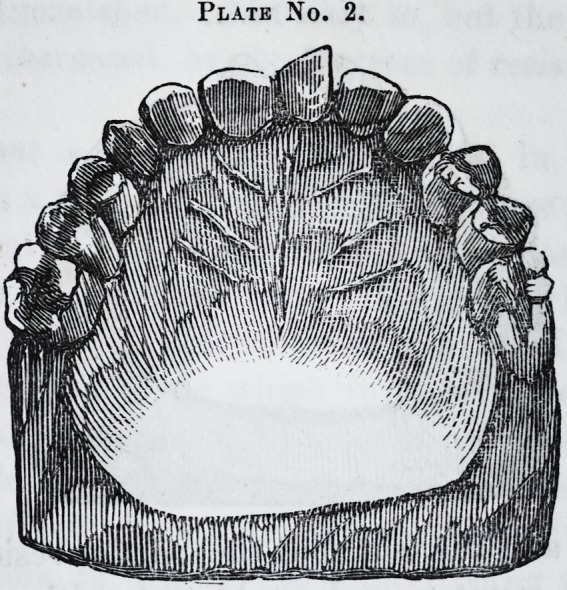# Irregularity

**Published:** 1859-07

**Authors:** S. M. Shepherd

**Affiliations:** Petersburg, Va.


### ARTICLE VII.

### *Irregularity.* By S. M. SHEPHERD, D. D. S.

THE views which I shall offer on this subject will be found to differ, in some degree, from those of some writers. The subject is one of great importance as well as prominence, inasmuch as it makes itself to be regarded not only by the profession, but by all classes. I take the ground that too much is frequently done to correct, by mechanical manipulation, what would be better done by nature, if nature were left alone to act. The strong solicitude of parents for their children, especially of mothers for their daughters, prompts them to observe the slightest malposition of their teeth, and to use all possible means for their speedy correction. When told by the dentist that the irregularity is slight, and that nature will correct it—that as the teeth advance they will naturally incline toward a correct position, they doubt it and are unwilling to risk it. They are willing, without scruple, to submit to ligatures. bands, plates, or what not, if the dentist thinks it is necessary, and it is generally less difficult to manipulate when his patrons demand it, than to maintain an adverse judgment to theirs. But the demand of the anxious mother is generally in harmony with the honest conviction of the dentist; the prevalent doctrine on the subject is, the sooner the better—that by delay the deformity is increased rather than diminished. Not only so, but the difficulty of correction is increased by the increase of resistance in the alveolus.

I admit that a tooth may be changed in its position more easily in a young subject than an old one; and I also admit that that there are some cases of mal-arrangement, which may be corrected in early childhood, which become impossible if neglected for a few years; and I also admit that there are some cases, which in the nature of things, grow worse from the first appearance of the teeth until they are fully developed, but they are not all so. There are many cases, which if left alone will correct themselves. There are many others in which the subject being very young, will grow no worse by prudent delay, and may be more easily corrected when the subject becomes older and the teeth more advanced.

Nature is the great rectifier of her own defects, and if we would exercise patience and wait on her operations, we should have much less to do in the end, than when we undertake to do all ourselves. Besides *perfect* regularity is not essential to beauty. A very slight deviation in position is an evidence of nature that is sometimes copied in art for effect.

The principle I advocate finds support in the following case:

About six years ago, Mrs. F. called on me, and presented her daughter, aged nine, and consulted me in reference to the left central incisor, which had come through the gum, exactly across the alveolar ridge, and presented the edge to the front, as in plate No. 1.

As will be observed, the tooth had not advanced far enough to admit of the attachment to it of any kind of a fixture to force it round; and then the molar and bicuspid teeth were only partially developed; so that a ligature would require to be completely imbedded in the gum to find a resting place for securing the other end of the lever. At best, I looked upon it as a case of extreme difficulty;

PLATE No. 1.

and to this, add the fact that the parties resided at a distance in the country, and could not, with any degree of convenience, visit my office as frequently as might be necessary. There was no ground to hope that nature would do anything for this case; if ever turned, it must be done by art; and in the present condition of the mouth, it could not even be commenced with propriety. I, therefore, advised that no active treatment should be adopted until after a lapse of one or two years, only that the mother should frequently take hold of it with a small pair of pliers, with which I furnished her, and gently twist it in the right direction. This she promised me she would do to the best of her ability, and call on me again at the appointed time.

Six years elapsed before I saw my little patient again. Instead of nine, she was now fifteen; and instead of a little girl, she had become quite a grown up young lady.

On examination of the mouth, I found it as represented in plate No. 2.

On inquiry, I learned that the instrument had been used very little, not enough, I should judge, to make any material difference; and yet the tooth had come round almost to its proper place; and what did it, if nature herself did not? Is it at all probable that the very slight twisting applied by the mother—a timid, fearful, and tender mother—could have effected it? Those who have

PLATE No. 2.

had any experience in regulating would scarcely think it did. Here then is a case least of all likely to be corrected by nature, where no actual obstruction intervenes, and yet the strong inference is, that nature did it to the very creditable extent to which it has been done, and may, in the next two, three, or four years, make a complete job of it. I would not make the impression, by what I have said, that I think it wrong to correct irregularities by artificial means, and even severe means in some cases; for there are very many cases that may be most happily corrected at the proper time by very slight means, which become difficult if neglected only a short time, and impossible if neglected long. But I am satisfied that children are often afflicted with a regulating apparatus, who if left to nature, would in time have as good, if not a better, arrangement of their teeth, than they get by artificial means.

It requires good judgment on the part of the dentist to enable him to determine, when a case presents itself, what is proper to be done. But there are cases of doubt, as the one which I have described, and I always prefer, in such cases, to do too little rather than too much.

S. M. SHEPHERD.

PETERSBURG, VA., *May* 6, 1859.

## Figures and Tables

**Plate No. 1. f1:**
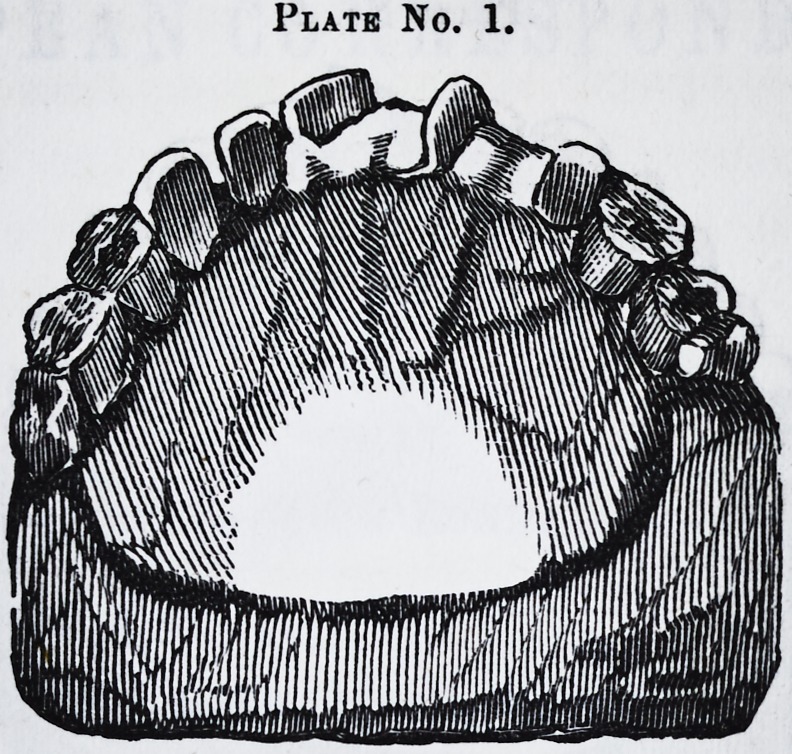


**Plate No. 2. f2:**